# Cemiplimab as First Line Therapy in Advanced Penile Squamous Cell Carcinoma: A Real-World Experience

**DOI:** 10.3390/jpm13111623

**Published:** 2023-11-20

**Authors:** Keren Rouvinov, Gal Mazor, Ella Kozlener, Amichay Meirovitz, Noa Shani Shrem, Omar Abu Saleh, Sondos Shalata, Alexander Yakobson, Walid Shalata

**Affiliations:** 1 The Legacy Heritage Center & Dr. Larry Norton Institute, Soroka Medical Center and Ben Gurion University, Beer Sheva 84105, Israel; 2 Medical School for International Health, Ben Gurion University of the Negev, Beer Sheva 84105, Israel; 3 Department of Oncology, Bnei Zion Medical Center, Haifa 31048, Israel; 4 Department of Dermatology and Venereology, Emek Medical Centre, Afula 18341, Israel; 5 Nutrition Unit, Galilee Medical Center, Nahariya 22000, Israel

**Keywords:** cemiplimab, penile carcinoma, chemotherapy-ineligible, squamous cell carcinoma, immune checkpoint inhibitors (ICIs)

## Abstract

In the treatment of cancer, immune checkpoint inhibitors (ICIs) have demonstrated significantly greater effectiveness compared to conventional cytotoxic or platinum-based chemotherapies. To assess the efficacy of ICI’s in penile squamous cell carcinoma (pSCC) we performed a retrospective observational study. We reviewed electronic medical records of patients with penile squamous cell carcinoma (SCC), diagnosed between January 2020 and February 2023. Nine patients were screened, of whom three were ineligible for chemotherapy and received immunotherapy, cemiplimab, in a first-line setting. Each of the three immunotherapy-treated patients achieved almost a complete response (CR) after only a few cycles of therapy. The first patient had cerebral arteritis during treatment and received a high-dose steroid treatment with resolution of the symptoms of arteritis. After tapering down the steroids dose, the patient continued cemiplimab without further toxicity. The other two patients did not have any toxic side effects of the treatment. To the best of our knowledge, this is the first real world report of near CR with cemiplimab as a first-line treatment in penile SCC.

## 1. Introduction

Penile cancer is a rare and aggressive neoplasm that accounts for less than 1% of male malignancies worldwide (0.84 per 100,000) but with high rates concentrated in the developing world (2–5.7 per 100,000) such as in Latin America and Africa, where neonatal circumcision is low and socioeconomic conditions predispose patients to multiple risk factors [1,2]. Risk factors for penile cancer SCC (pSCC) include the absence of childhood circumcision, phimosis, chronic inflammation, poor penile hygiene, smoking, immunosuppression, lichen sclerosus and infection with human papillomavirus (HPV) [1,3,4,5].

Guidelines recommend treatment for advanced disease with chemotherapy, using cisplatin and taxane-based regimens. The chemotherapeutic treatment for advanced penile squamous cell carcinoma has substantial side effects and no randomized data sup-port an overall survival benefit. There is a need for the development of more effective and less toxic therapeutic options [6,7]. Immune checkpoint inhibitors (ICI’s) have been shown to improve survival in a wide range of diseases, with a meaningful effect on the overall survival of patients [8,9]. There is a recent retrospective trial that demonstrated the efficacy of immunotherapy in advanced penile carcinoma patients, but only 18 patients were treated in the frontline setting [9]. It has been shown that there is high PD-L1 expression and a high level of CD8+ T-cell infiltration in penile cancer, which makes it a good candidate for immunotherapy with checkpoint blockade with a good chance of immunotherapy response in the treatment of locally advanced disease [10,11]. Cemiplimab is a humanized IgG4 monoclonal antibody designed to specifically target PD-1 (Programmed Cell Death Protein 1). Notably, this antibody, cemiplimab, does not activate antibody-dependent cellular cytotoxicity. It has received FDA approval for the treatment of several types of cancer, including basal cell carcinoma, non-small cell lung cancer (NSCLC) and cutaneous squamous cell carcinoma (cSCC). In patients receiving cemiplimab, the most commonly reported immune-related adverse events (irAEs) include hyperthyroidism, pneumonitis, nephritis, pruritus (itching), hepatitis, colitis, rash and hypothyroidism [12,13,14]. Two anti-PD-1 ICIs have shown preliminary evidence of activity: nivolumab showed a promising response in one patient with chemo-radiation refractory advanced penile cancer and pembrolizumab showed a durable response in two cases with chemo-radiation refractory metastatic penile squamous cell carcinoma [15,16].

Here, we report a retrospective observational study for patients with SCC of the penis showing response to the anti-PD-1 ICI cemiplimab in a first-line setting.

## 2. Materials and Methods

### 2.1. Selection of Patients

This retrospective non-interventional observational study was conducted across two institutions. The study cohort comprised all patients diagnosed with pSCC who received immunotherapy (IO) as their treatment between January 2020 and February 2023. The last follow-up date for data collection was 6 August 2023. The data collected encompassed details regarding the treatment regimen, commencement and conclusion dates of therapy, the date of the last follow-up, recorded toxicities, as well as overall survival (OS) and progression-free survival (PFS) durations. Ethical approval for this study was granted by the Institutional Review Board of Soroka Medical Center (approval no. 0189, granted on 27 June 2022) and Bnai Zion Medical Center, which granted a waiver due to the study’s single-case nature.

### 2.2. Clinical Data

#### 2.2.1. Patient Data Collection

We collected comprehensive patient data, including treatment details, therapy commencement and conclusion dates, last follow-up date, mortality date, disease progression date, overall response rate (ORR) and treatment-related toxicities. Additionally, we recorded performance status (PS) evaluations and detailed medical and therapeutic histories and Eastern Cooperative Oncology Group (ECOG).

#### 2.2.2. Treatment Response Evaluation

Our assessment of treatment responses focused on measurable target lesions, encompassing observable pSCC lesions noted during follow-up or assessable lesions detected through radiological imaging. Treating oncologists evaluated treatment responses using the Immune-Related Response Evaluation Criteria in Solid Tumors (iRECIST), categorizing responses into complete response (CR), partial response (PR), stable disease (SD) and progressive disease (PD). The disease control rate (DCR) was calculated based on patients achieving CR, PR or SD. Clinical or Radiological responses, such as clinical/radiological CR (c/rCR), clinical/radiological PR (c/rPR), clinical/radiological SD (c/rSD) and clinical/radiological PD (c/rPD), were determined through radiological follow-up or through physical examinations. Safety profiles were assessed using the Common Terminology Criteria for Adverse Events (CTCAE) version 5.0 [17].

#### 2.2.3. Pre-Treatment Evaluations

Before treatment initiation, patients underwent disease staging through total body computed tomography (CT) scans or fluorodeoxyglucose (FDG) positron emission tomography–computed tomography (PET-CT). Baseline laboratory tests assessed organ functions and parameters, including complete blood cell counts, renal and liver function markers, albumin levels, alkaline phosphatase levels and thyroid function markers. Viral infection tests for hepatitis B, hepatitis C and human immunodeficiency virus (HIV) were also conducted.

#### 2.2.4. Monitoring

Throughout each treatment cycle, routine laboratory tests were performed. For patients not requiring reassessment for certain infections, specific tests were omitted in subsequent treatment cycles. In addition, follow-up radiologic reevaluations (CT or FDG-PET-CT) were repeated every two to three months.

### 2.3. The Inclusion Criteria

Mature patient (patients should be 18 years of age or older).

Histologically confirmed pSCC.

Initial treatment with IO (immunotherapy) because of cisplatin or chemotherapy ineligibility.

Performance status (ECOG) score ranging from 0 to 4.

No prior systemic therapy for advanced or metastatic disease.

Treatment received at Soroka and Bnai Zion Medical Centers or complete follow-up history available in their records.

Each study patient underwent evaluation by a multidisciplinary medical team upon admission to Soroka and Bnai Zion Medical Center Oncology Institutes, as per the standard protocol. This team consists of dermatologist, medical oncologists, radiation oncologists, a pathologist, an imaging and nuclear physician and a plastic surgeon. Their discussions are based on patients’ pathological status, imaging status and performance status. A primary physician is assigned to oversee each patient’s treatment.

Each patient that was diagnosed with advanced or metastatic disease (any T, N 1-3 and/or M1) is primarily managed by medical oncologists, following National Comprehensive Cancer Network (NCCN) recommendations [18].

**Figure 1:**
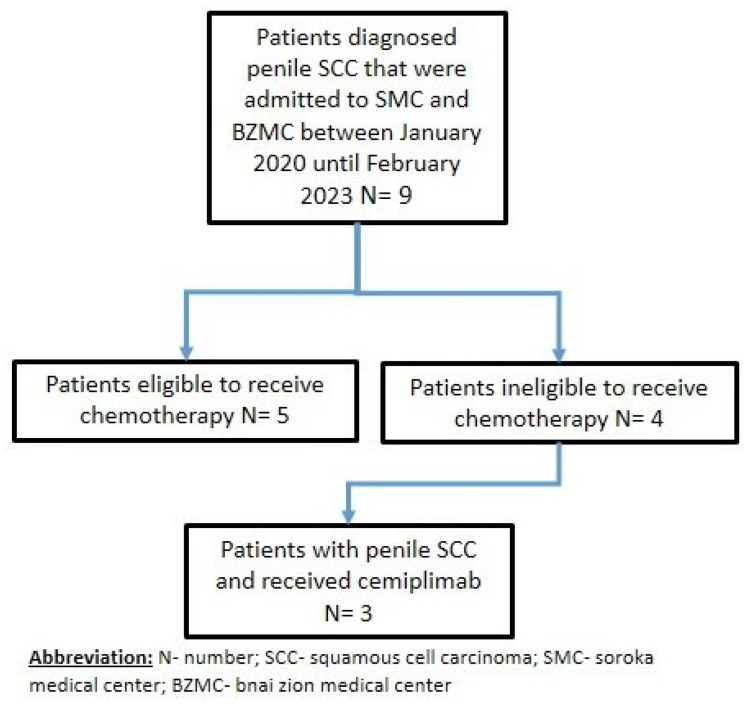
The workflow used for the two institutions, retrospective, observational study of penile squamous cell carcinoma treated with cemiplimab.

Out of 9 screened patients, 4 met the eligibility criteria for this study. Unfortunately, one of these four eligible patients, who was undergoing dialysis for renal failure, passed away from sepsis after the first cycle of cemipimab. As a result, his response status could not be evaluated (Figure 1).

### 2.4. Exclusion Criteria for the Study

Patients with autoimmune diseases.

Patients undergoing immunosuppressive therapy (such as steroids, methotrexate).

Patients who had received previous immunotherapy treatment or any systemic therapy for SCC within the past year were excluded from the study to ensure more accurate results that were not influenced by the previous therapy.

## 3. Results

### 3.1. Elderly Patient with a Chronic Renal Failure

A male aged 77 years, who has a medical history of diabetes type 2, including retinopathy, nephropathy, diabetic foot, ischemic heart disease, atrial fibrillation, hypertension and chronic renal failure, with a creatinine level of 2.4 mg/dL (normal 0.67–1.17 mg/dL). He was a non-smoker with a family history of a brother with colon cancer and a father with gastric cancer.

**Figure 2:**
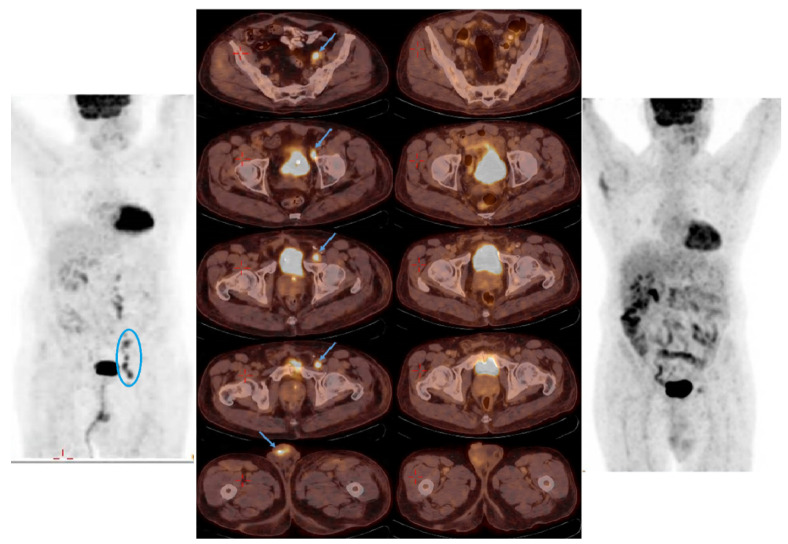
FDG-PET-CT scan revealed the metastatic disease (left figures), (blue arrows and circle indicates metastasis), showing increase in metabolic absorption in the glans of the penis; increase metabolic absorption and enlargement of the lymph nodes in the left retroperitoneal area; and increase in metabolic absorption in the pelvic and inguinal nodes. The right-side figures show the FDG-PET-CT scan with a significant response and the complete radiological regression of the lesions.

In November 2021, he was referred from the urology department after the diagnosis of squamous cell carcinoma of the penis. Two months before his diagnosis, an ulcerated lesion appeared on the head of the penis. The patient was consulted by a dermatologist and an urologist and was recommended for circumcision. Pathological results confirmed the diagnosis of squamous cell carcinoma of the penis. Physical examination, including lung and cardiovascular evaluation, showed no pathological abnormalities. Echocardiography was normal with no pathological findings. Routine laboratory investigation (full biochemistry profile and complete blood count (CBC)) was within normal ranges, except for an elevated creatinine level of 2.5 mg/dL (normal 0.67–1.17 mg/dL). An FDG-PET-CT scan in December 2021 showed increased metabolic absorption with high intensity in the glans of the penis, and multiple metabolic absorption lymph nodes in the retro-abdominal space, mainly on the left retroperitoneal, pelvic and inguinal node areas (Figure 2), The pathological stage was identified as T3-N2-M0 (stage IIIB).

In addition, the patient underwent tests for HPV and human immunodeficiency virus (HIV), which were negative.

A multidisciplinary team, including a radiologist, urologist and oncologist, considering the patient’s age, performance status and medical history including chronic renal failure, recommended immunotherapy with cemiplimab. After receiving two cycles of therapy, there was a decrease in the size of the lymph nodes with an improvement of the lesions in the penis. After receiving five cycles of cemiplimab, the patient was hospitalized with temporal arteritis, which was confirmed by biopsy, and received high-dose steroid treatment (60 mg). Immunotherapy was resumed after tapering down the steroids and the resolution of all symptoms. A repeated FDG-PET-CT scan from September 2022 showed complete radiological response of the lesions and all lymphatic nodes. As of the most recent follow-up (July 2023), the patient is still on treatment and has complete radiological response (Figure 2).

### 3.2. Eligible for Cisplatin but Refusing Surgery and Chemotherapy

A male aged 73 years, who has a medical history of chronic ischemic heart disease and hypertension. The patient was a smoker (30 packs/year).

Due to an enlarged inguinal lymph node (approximately 3 cm) and a large lesion on the glans of the penis, in January 2022 he was referred to a urologist for a consultation by his family physician. According to the patient, he noticed the changes approximately four months before the urologist appointment. In January 2022 after the urologic consultation, he underwent a biopsy of the glans of the penis and the pathological results confirmed the diagnosis of pSCC. Subsequently, the patient underwent tests for HPV and human immunodeficiency virus (HIV), which were negative, and he was referred for oncological consultation.

**Figure 3:**
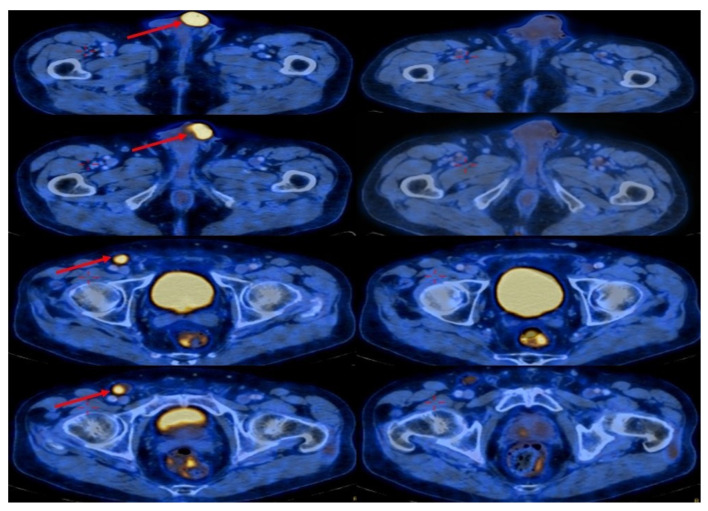
FDG-PET-CT scan revealed the metastatic disease (left figures), (red arrows indicates metastasis), showing increase in metabolic absorption in the glans of the penis; increased metabolic absorption; and enlargement of the lymph nodes in the right inguinal area. The right-side figures show the FDG-PET-CT scan with a significant radiological response and the complete eradication of the lesions.

The FDG-PET-CT scan from February 2022 showed no signs of metastatic spread, except for an increased metabolic absorption in the penis with right groin lymphadenopathy (Figure 3), The pathological stage was identified as T3-N1-M0 (stage IIIA).

A multidisciplinary team, including a radiologist, urologist and oncologist recommended the patient undergo radical surgery but the patient refused surgery or chemotherapy. Therefore, systemic immunotherapy with cemiplimab was initiated, and he started his first cycle in March 2022.

During his treatment, a routine laboratory investigation (CBC and full biochemistry profile) revealed no abnormalities. An FDG-PET-CT scan 4 months later (June 2023) showed a significant reduction in the metabolic absorption of the skin lesion of the penis and a reduction in the metabolic absorption of the right inguinal lymph node. In last follow-up (July 2023) the patient was still under treatment and in complete radiological response (Figure 3).

### 3.3. Chronic Renal Failure with Extensive Disease

A male aged 83 years, who has a medical history of ischemic heart disease, a history of coronary artery bypass grafting in 2021 with left ventricular ejection fraction 43%, hypertension, chronic renal failure and diabetes type 2. The patient reported 5 kg weight loss during the last 3 months.

According to the patient, ulcerated lesions appeared on the inferior part of the penis about two months before his consultation with the urologist. He underwent biopsies from those lesions and the pathological results confirmed the diagnosis of SCC of the penis. The physical examination, including lung, cardiovascular and abdominal evaluation, was normal. A routine laboratory investigation (CBC and full biochemistry profile) showed no abnormalities except chronic renal failure with a creatinine level of 3.1 mg/dL (normal 0.67–1.17 mg/dL). The patient underwent tests for HPV and human immunodeficiency virus (HIV), which were negative and he was referred for oncological consultation.

An FDG-PET-CT scan from January 2023 showed increased metabolic absorption with high intensity in the penis, perineum and scrotum and multiple metabolic absorption of the lymph nodes in the right inguinal region and bilateral iliac nodes, the pathological stage was identified as T3-N3-M0 (stage IV). These findings suggested an extensive tumor process and the involvement of pelvic and inguinal lymph nodes.

A meeting involving multiple disciplines, including a radiologist, urologist and oncologist, considering the patient’s age and the extent of disease, recommended the patient be treated with systemic immunotherapy with cemiplimab. After the first cycle, a significant reduction in the size of the tumor and penis lesions was noted on visual examination.

## 4. Discussion

In the current study we have provided descriptions of patients diagnosed with advanced or metastatic penile squamous cell carcinoma (pSCC). This type of cancer is recognized for its rarity which comprises less than 1% of male malignancies worldwide [1,2]. SCC accounts for the majority of cases of penile cancer [19]. The exact cause of penile cancer remains incompletely understood. pSCC can originate from penile intraepithelial neoplasia (PIN) or arise spontaneously. High-risk HPV infections, particularly strains 16 and 18, have been linked to pSCC. In addition, approximately 30–50% patients with penile cancer are HPV positive 17 and 32–67% are PD-L1 positive [20,21,22,23].

HPV likely promotes penile cancer development through its viral oncogenes E6 and E7, which are actively expressed by HPV-infected cells. E6 targets the p53 gene, while E7 targets RB1; both p53 and RB1 are tumor suppressor genes that normally inhibit cell proliferation. When these genes are altered, uncontrolled cell growth may occur, leading to malignancy. HPV DNA has been detected in only 22–72% of pSCC cases, whereas it is found in the majority of PIN cases (70–100%). This disparity in HPV DNA presence between SCC and PIN suggests a multifactorial etiology, with both HPV-independent and HPV-dependent pathways contributing to the development of pSCC [20,21,22,23].

Two of our patients were ineligible to receive cisplatin as a first-line treatment due to renal failure and the third received treatment as the “patient’s choice” due to refusing to undergo surgery or chemotherapy; therefore, it was decided to treat them with cemiplimab, which showed very impressive results. In 2018, the FDA approved cemiplimab for the treatment of locally advanced and metastatic cutaneous SCC (cSCC) [12,13,14,24]. Cemiplimab is a monoclonal antibody of the immunoglobulin-G class that binds to the programmed cell death protein 1 receptor on T cells, which prevents the inactivation of T cells by the tumor cell receptor ligands programmed death ligand 1 and programmed death ligand 2 [12,13,14,25]. By inhibiting the inactivation of T cells by tumor cells, T cells are able to trigger the apoptosis of tumor cells. While other monoclonal antibodies that target programmed cell death protein 1 exist, cemiplimab was the first antibody to be approved for cSCC [23]. The FDA approved this drug based on a study that included 108 patients with advanced cSCC. The overall response rate for those with metastatic cSCC was 47%, while for those with locally advanced cSCC it was almost 50%. Additionally, 61% of the responses lasted for 6 months or more. Given the high response rates observed in patients with unresectable or metastatic cSCC, there are ongoing clinical trials to investigate the potential of neo-adjuvant cemiplimab in the treatment of resectable cSCC [26].

Little is known about the efficacy of cemiplimab, or immunotherapies in general, as a first-line treatment for pSCC. A recently published article in the ASCO GU 2023 presented retrospective data which showed the efficacy of immunotherapy in locally advanced and metastatic pSCC [9]. Among that cohort were 92 patients, of which the median age was 62 years (53–70). Notably, 83 patients (90%) were diagnosed with metastatic penile squamous cell carcinoma and the majority of the patients (80%) had undergone at least second-line treatments. The overall response rate (ORR) of 13%. Stable disease was seen in 28% of patients [9]. The median PFS was 3.2 months and the median OS was 9.8 months. Notably, among patients with lymph node-only metastases, the response rate was higher at 35%, with 7 out of 20 patients showing a positive response. Eighteen patients were treated with immunotherapy in the first line setting, with a disease control rate of 47%. No complete response was seen. Patients were treated with different immunotherapy drugs: ICIs monotherapy (28% pembrolizumab, 17% nivolumab, 16% cemiplimab, 13% other) or combination (12% ipilimumab plus nivolumab or 13% ipilimumab plus nivolumab and cabozantinib). ORR with ICI monotherapy was only 8.5% [9] It was noted that several factors were identified as indicators of worse overall survival, including the presence of visceral metastases, an ECOG performance status score of 1 or higher and a higher neutrophil-to-lymphocyte ratio [9]. Furthermore, in previously published meta-analyses and articles, it has been mentioned that patients with various diagnoses, who were treated with immunotherapy as the first line of therapy, have demonstrated better outcomes in terms of OS, PFS, and ORR compared to those who received it as a second-line treatment or in advanced lines [27,28,29,30,31,32,33]. Therefore, upon comparing our results with the previously mentioned cohort, we have reached the conclusion that administering a PD-1 inhibitor, specifically cemiplimab, as a first-line therapy yields better outcomes than in patients who received PD-1 therapies in more advanced treatment lines.

Patients diagnosed with pSCC who had chronic renal failure or were ineligible for cisplatin or chemotherapy were not included in these studies. Consequently, there is limited information available regarding the management of such complex scenarios that are commonly encountered in real-world clinical practice [9,15,16]. In our cohort, the observed response surpassed our initial expectations and existing knowledge, yielding noteworthy and substantial results. One potential explanation for this outcome is the retrospective studies suggesting potential advantages of immunotherapy in elderly patients compared to their younger counterparts [13,34,35,36]. This observation may be associated with a more favorable antitumor balance, characterized by higher CD8 T cell activity relative to regulatory T cells within the tumor microenvironment [13,34,35,36]. In this context, we present the efficacy and emphasize the paramount importance of real-world data in pSCC. The significance of such data cannot be overstated, especially when considering the diverse and often medically frail baseline characteristics observed in this patient population.

HPV status was not a predictor of PFS (*p* = 0.87) or OS (*p* = 0.73) in data presented by T. El Zarif [9]. Patients with a neutrophil lymphocyte ratio (NLR) <5 and an absence of visceral metastases showed better OS (*p* = 0.0054) [9]. There is, to the best of our knowledge, no published data regarding the correlation between PD-L1 and the response to ICI in patients with advanced penile cancer. The therapeutic efficacy of immunotherapy on tumors supports the ongoing inquiry of whether the early onset of immune-related adverse events could serve as a predictive indicator of enhanced tumor response to immunotherapy [37,38,39,40,41].

Recently, it was mentioned that also elderly (over 75 year old) and immunocompromised patients with advanced or metastatic cutaneous SCC that received cemiplimab had good results, compared with real world data [13].

In the complex environment of tumors, PD-1 and its counterpart, PD-L1, play a pivotal role in promoting tumor growth and survival while evading immune surveillance. PD1, classified as a checkpoint protein and a member of the CD28 family, belongs to a group of inhibitory T-cell receptors that are not constitutively expressed but rather upregulated following antigen stimulation and cytokine signals produced during T cell activation. Beyond T cells, PD1 is also expressed in B cells, monocytes and dendritic cells (DCs), where it modulates various aspects of immune function. On the other hand, PDL1, a type 1 transmembrane glycoprotein within the B7 ligand family, is not limited to activated T and B cells; it is also found on certain non-hematopoietic cells, particularly antigen-presenting cells like DCs. When T cells recognize tumor cells and aim to eliminate them, tumor cells respond by upregulating the PDL1 protein, which in turn binds to PD1 on T cells, ultimately leading to T cell apoptosis.

The presence of PDL1 on the surface of tumor cells can be heightened by interferon-gamma (IFN-γ) produced by activated T cells. This PD1/PDL1 signaling pathway constitutes a crucial element in tumor-associated immunosuppression, inhibiting T lymphocyte activation and reinforcing the immune tolerance of tumor cells, enabling them to evade the immune system. To sum up, the binding of PD1 to PDL1 serves to diminish T cell-mediated immune surveillance, resulting in an absence of immune response and even triggering T cell apoptosis. This interaction also hampers the function of tumor-infiltrating CD4+/CD8+ T cells (CD4+/CD8+ TILs) and reduces the production of cytokines such as tumor necrosis factors, IFN-γ and Interleukin-2. This, in turn, provides cancer cells with an avenue to evade the immune response. In contrast, PD1/PDL1 inhibitors disrupt the immunosuppressive effects on anti-tumor T cells, leading to increased T cell proliferation and infiltration into the tumor microenvironment, thereby triggering an anti-tumor response. Current anti-PD1/PDL1 therapies block the interaction between PD1 and PDL1, effectively reactivating suppressed immune cells and initiating an anti-tumor immune response [13,41,42,43,44,45,46,47,48,49,50,51,52]. The ICIs function by obstructing the inhibitory signals originating from tumor cells to the T cells that target them. Consequently, blocking the PD-1/PD-L1 interaction using antibodies enhances immune responses directed at tumor cells. Moreover, this approach has demonstrated effectiveness in treating various forms of cancer and has been documented to enhance outcomes, such as overall survival (OS) and progression-free survival (PFS), in several diagnoses [27,28,29,30,31,32,33,52,53,54,55,56,57,58,59,60,61].

The recommended therapy for patients with advanced penile carcinoma is chemotherapy with a cisplatin- and taxane-based regimen. It is important to note that some patients are ineligible for cisplatin and little is known about carboplatin-based combinations.

Our study has several limitations that should be considered. A limitation of this study is that it was retrospective study. One notable limitation is that our analysis relied solely on data obtained from two institutions. To enhance the robustness and generalizability of these findings, it is essential for future research to expand its scope. This can be achieved by incorporating data and informations from several centers or even multiple countries, in addition, by including a larger cohort of patients in that analysis.A broader and more diverse dataset can provide a more comprehensive and reliable basis for drawing conclusions and making meaningful clinical recommendations; on the other hand, pSCC is a very rare type of cancer. Furthermore, the inclusion criteria of this study allowed only patients ineligible for cisplatin. Therefore, future investigations and studies involving data gathered from numerous centers and countries, featuring more extensive patient cohorts and using different types of ICIs, should be carried out to validate these findings and results. Our recommendation is to initiate prospective trials for advanced or metastatic pSCC with IO as first line of treatment with the use of multidisciplinary patient management. In addition, additional research on finding predictive biomarkers is needed to identify pSCC patients that benefit the most from ICIs.

Even though our investigational study had inherent limitations, it holds significant importance in that it is representing, to the best of our knowledge, an initial attempt to characterize the effectiveness and the safety of IO in patients diagnosed with pSCC. Particularly in those who are ineligible to receive cisplatin or choose not to pursue the surgical option. The insights and data we have gathered in this study could carry substantial significant implications for the treatment and care of this particular group of patients. This pioneering research may pave the way for new treatment approaches and strategies that can better address the needs and challenges faced by these patients in their battle against pSCC.

## 5. Conclusions

In our article, efficacy was shown with cemiplimab as a first-line treatment in patients who were ineligible or refused combined cisplatin-based chemotherapy or surgery. Our patients achieved almost complete radiological response. Undoubtedly, radical surgery or radiotherapy will remain an important part of the treatment strategy for responding patients and remain the standard of care for localized pSCC, but the use of IO will certainly reinforce the need to develop predictive biomarkers.

## Data Availability

No new data were created or analyzed in this study. Data sharing is not applicable to this article.
